# 76 Resuscitation Volumes Affect Perfusion and Inflammatory Cytokine Expression in Peri-Burn Skin: Implications for Burn Conversion

**DOI:** 10.1093/jbcr/irad045.050

**Published:** 2023-05-15

**Authors:** Edward Kelly, Bonnie Carney, Eriks Ziedins, Babita Parajuli, David Burmeister, Lauren Moffatt, Jeffrey Shupp

**Affiliations:** Firefighter's Burn and Surgical Research Laboratory, Washington, DC; Firefighters' Burn and Surgical Research Laboratory, Medstar Health Research Institute, Georgetown University Medical Center, Washington, DC; Firefighters' Burn and Surgical Research Laboratory, Washington, DC; Uniformed Services University of the Health Sciences, Bethesda, Maryland; Uniformed Services University of the Health Sciences, Bethesda, Maryland; Medstar Health, Washington , DC; Firefighters' Burn and Surgical Research Laboratory, MedStar Health Research Institute, Georgetown University School of Medicine, Washington, DC; Firefighter's Burn and Surgical Research Laboratory, Washington, DC; Firefighters' Burn and Surgical Research Laboratory, Medstar Health Research Institute, Georgetown University Medical Center, Washington, DC; Firefighters' Burn and Surgical Research Laboratory, Washington, DC; Uniformed Services University of the Health Sciences, Bethesda, Maryland; Uniformed Services University of the Health Sciences, Bethesda, Maryland; Medstar Health, Washington , DC; Firefighters' Burn and Surgical Research Laboratory, MedStar Health Research Institute, Georgetown University School of Medicine, Washington, DC; Firefighter's Burn and Surgical Research Laboratory, Washington, DC; Firefighters' Burn and Surgical Research Laboratory, Medstar Health Research Institute, Georgetown University Medical Center, Washington, DC; Firefighters' Burn and Surgical Research Laboratory, Washington, DC; Uniformed Services University of the Health Sciences, Bethesda, Maryland; Uniformed Services University of the Health Sciences, Bethesda, Maryland; Medstar Health, Washington , DC; Firefighters' Burn and Surgical Research Laboratory, MedStar Health Research Institute, Georgetown University School of Medicine, Washington, DC; Firefighter's Burn and Surgical Research Laboratory, Washington, DC; Firefighters' Burn and Surgical Research Laboratory, Medstar Health Research Institute, Georgetown University Medical Center, Washington, DC; Firefighters' Burn and Surgical Research Laboratory, Washington, DC; Uniformed Services University of the Health Sciences, Bethesda, Maryland; Uniformed Services University of the Health Sciences, Bethesda, Maryland; Medstar Health, Washington , DC; Firefighters' Burn and Surgical Research Laboratory, MedStar Health Research Institute, Georgetown University School of Medicine, Washington, DC; Firefighter's Burn and Surgical Research Laboratory, Washington, DC; Firefighters' Burn and Surgical Research Laboratory, Medstar Health Research Institute, Georgetown University Medical Center, Washington, DC; Firefighters' Burn and Surgical Research Laboratory, Washington, DC; Uniformed Services University of the Health Sciences, Bethesda, Maryland; Uniformed Services University of the Health Sciences, Bethesda, Maryland; Medstar Health, Washington , DC; Firefighters' Burn and Surgical Research Laboratory, MedStar Health Research Institute, Georgetown University School of Medicine, Washington, DC; Firefighter's Burn and Surgical Research Laboratory, Washington, DC; Firefighters' Burn and Surgical Research Laboratory, Medstar Health Research Institute, Georgetown University Medical Center, Washington, DC; Firefighters' Burn and Surgical Research Laboratory, Washington, DC; Uniformed Services University of the Health Sciences, Bethesda, Maryland; Uniformed Services University of the Health Sciences, Bethesda, Maryland; Medstar Health, Washington , DC; Firefighters' Burn and Surgical Research Laboratory, MedStar Health Research Institute, Georgetown University School of Medicine, Washington, DC

## Abstract

**Introduction:**

Fluid resuscitation after thermal injury is paramount to avoiding the effects of burn shock and restoring organ perfusion. Both over and under resuscitation can lead to unintended consequences affecting patient outcomes. While many studies have focused on the systemic effects of resuscitation, there is limited information on how fluid resuscitation might affect the burn wound, specifically burn wound progression, in the acute period. Furthermore, the mechanisms underlying burn wound progression are not fully understood. For these reasons, a polytrauma swine model was used to investigate varying levels of resuscitation on burn wound dynamics.

**Methods:**

Nine female Yorkshire pigs were used in this experiment. Pigs were anesthetized and subjected to 40% total body surface area burn and 15% hemorrhage. Pigs were randomized (n=3) to receive different resuscitative strategies: decision support driven (adequate, 2-4ml/kg/%TBSA), fluid withholding (under, < 2ml/kg/%TBSA) or high constant rates (over, > >4ml/kg/%TBSA). Pigs were then monitored for 24hrs in an intensive care setting prior to necropsy. Laser Doppler Imaging (LDI) was conducted over the burn wound and peri-wound skin at pre-determined timepoints to assess perfusion. Skin biopsies were also taken from the burn, peri-burn (within 2cm of burn) and normal skin areas. RNA was isolated from normal skin and peri-burn skin biopsies at hour 6 and qRT-PCR was conducted to assess levels of common inflammatory cytokines: interlukein-6 (IL-6), chemokine CXC motif ligand-8 (CXCL8), and interferon-gamma (IFN-y).

**Results:**

At hour 2, LDI analysis demonstrated increased perfusion in the peri-burn skin of over-resuscitated animals when compared to adequate and under-resuscitated groups (p=0.002 and p=0.007, respectively). At hour 6, peri-burn skin of over-resuscitated animals showed increased perfusion when compared to the adequate resuscitation groups (p=0.01). At hour 6, peri-burn skin samples showed increased expression of all 3 cytokines in adequately resuscitated animals when compared to over and under resuscitated groups (IL-6: 2.94 vs. 1.47 and -0.11-fold, CXCL8: 9.04 vs. 0.82 and -0.31-fold, IFN-g: 2.09 vs. -0.48 and -0.81-fold).

**Conclusions:**

This animal model shows differences in both perfusion and inflammatory cytokine expression in peri-burn skin samples based on resuscitation strategy. Varying levels of resuscitation following burn can have wide ranging consequences that may affect burn wound progression.

**Applicability of Research to Practice:**

Under or over-resuscitation may lead to local changes in the burn wound that could affect the overall severity and evolution of the injury over time. Judicious fluid resuscitation may not only be useful in dampening the possible systemic complications associated with fluid creep (eg. compartment syndrome), but may have direct effects on the local wound bed. By further elucidating mechanisms for burn conversion, interventions may be developed.

